# Commentary: Accessing 3D Printed Vascular Phantoms for Procedural Simulation

**DOI:** 10.3389/fsurg.2022.910447

**Published:** 2022-06-17

**Authors:** Som P. Singh, Fahad M. Qureshi, Farhan Baig

**Affiliations:** Department of Biomedical Sciences, University of Missouri - Kansas City School of Medicine, Kansas, MO, United States

**Keywords:** 3D printing, imaging, vascular, education, training

A Commentary on:

**Accessing 3D Printed Vascular Phantoms for Procedural Simulation Surgery** *by Coles-Black J, Bolton, D., and Chuen, J. (2021). Front Surg. 7:626212. doi: 10.3389/fsurg.2020.626212*

## Introduction

Vascular disease remains a global health concern despite continued innovation in its diagnosis and management (1). Given the rise in endovascular therapeutic intervention options for patients with vascular pathologies, there has been a tremendous improvement in postoperative outcomes throughout diagnosis patterns (2, 3). A primary factor behind the improved postoperative outcomes is due to the minimally invasive nature of such procedures which allow for less metabolic strain for patients during the recovery process (4). In particular, abdominal aortic aneurysms (AAA) - a pathological dilation of the abdominal aorta - have experienced statistical decreases in morbidity and mortality rates among patients who undergo endovascular repair when compared to open procedures (4, 5). Let alone, the innovation behind AAA endovascular repair has led to a growth in these therapeutic modalities used in emergent settings as well (6). However, like any therapeutic modality, the risk of developing complications still exists. For example, injuries to the access vessels have the potential for development of other complications such as vessel rupture, occlusion, and possible need for additional acute vascular interventions to repair the complications (2). These risks make it imperative for the healthcare field to continue to seek options to evaluate and innovate the continuum of medical training of interventionists and vascular surgeons for the goal of optimal patient outcomes. In particular, the aim of this commentary is to examine 3D printed phantoms as an innovation in vascular surgery.

## Application of 3D Printed Models in Preoperative Care

Historically, 3D printing was developed as a form of additive manufacturing in the 20th century. Initially, the use of 3D printing was isolated to industrial purposes. However, 3D printing has created an indelible impact in healthcare in the form of anatomical models for medical training, implantable prothesis, and surgical preparation through image guidance in an array of surgical subspecialties including vascular, orthopedic, and urological surgeries (6–17). Moreover, the use of patient-specific surgical models has led to the rise in use of these additive manufacturing modalities for improving surgical planning since its initial investigation in the 1990s. These models allow for ample time for proceduralists to prepare for potential complex anatomy which may not be initially observed through more common 2D imagining modalities. In addition, 3D printed models afford trainees to grow a repository of procedural simulations which amplifies the total exposure per trainee through these artificial models in addition to their clinical experiences. Ultimately, the primary benefit of this preparation is to improve workflow efficiency as discussed in previous literature (18, 19). In fact, this workflow efficiency has been shown to save greater than $1000 per case due to reduced operating room time requirements (20).

## 3D Printed Vascular Phantoms

In a recent methods article by Coles-Black et al. in 2021, the authors of this article developed a workflow methodology of developing 3D printed vascular phantoms from CT angiograms (18). This study introduced a thorough and efficient protocol that could potentially be translated into other healthcare practices given greater availability of 3D printing resources over time. Additionally, a key emphasis provided in the article was in description of the utility of patient-specific CT angiography, and the vested interest in the models which have a more complex anatomy at baseline which can contribute to improving surgical decision making. Moreover, the use of these 3D vascular phantoms can benefit vascular trainee confidence through simulation practice as discussed. Additionally, the authors discussed the potential for 3D printing services based in hospitals itself to provide a centralized process for project implementation.

## Application of 3D Printed Models in Medical Education

The methodology proved by Coles-Black et al. provides a foundation for further exploration in the utility of these models in healthcare. In addition, this commentary would like to build upon this discussion by introducing the potential for 3D printed vascular phantoms for the utilization of medical school curriculum. This furthers the educational potential of 3D vascular models to grow beyond vascular surgery training. Moreover, the medical student training proposal benefits include a focus on introducing complex anatomy and developing greater understanding on the nuances of how imaging modalities can function (19–21). 3D printed models can be embedded into the curriculum of medical education within preclinical years (often the first-half of medical school years), which can allow for better anatomic visualization. Moreover, the literature on the effects of 3D visualization programs in student curriculum has shown promise in potentially improving memory recall of anatomy and spatial understanding (18–24). However, the concerns regarding cost of implementation may serve as a potential barrier towards widespread implementation in medical student curriculum (23–25). This provides a further direction for future studies to investigate the cost-benefit analysis of implementing such a curriculum design. Furthermore, the utility of 3D printing implementation in school curriculum has been widely investigated in developing pathology curriculum as well. This evidence may further support the idea of its implementation in a medical school curriculum.

## Conclusion

3D vascular phantoms and the overall concept of 3D printing has numerous capabilities in healthcare and medical training. In this commentary, the primary areas of examination were on its utility in presurgical planning as seen in a variety of surgical subspecialties as well as in medical education. With regard to medical education, there may be potential utility in implementing vascular phantoms and 3D printing curriculum in medical school education as well. Future directions ought to include a greater degree of 3D printed vascular phantoms in medical care.
